# The Effect of Digoxin on Mortality and Morbidity in Patients With Chronic Heart Failure: A Secondary Survival Analysis of the Digitalis Investigation Group (DIG) Trial

**DOI:** 10.7759/cureus.113002

**Published:** 2026-07-20

**Authors:** Vishal P Desai, Jay P Desai, Poojaben Patel, Sonal Chauhan

**Affiliations:** 1 Epidemiology, Saint Louis University, St. Louis, USA; 2 Accountable Care Organization (ACO), Essen Health Care, New York, USA; 3 Medicine, Gujarat Adani Institute of Medical Sciences, Bhuj, IND; 4 Health Home, Essen Health Care, New York, USA

**Keywords:** chronic heart failure, digoxin, dig trial, epidemiology, hospitalization, kaplan-meier, morbidity, mortality, survival analysis, worsening heart failure

## Abstract

Background

The long-term efficacy and safety of cardiac glycosides in the treatment of chronic heart failure with normal sinus rhythm remain topics of clinical debate. This study assessed the impact of digoxin on overall mortality and hospitalization rates in patients with heart failure and reduced ejection fraction. This is a retrospective secondary survival analysis of the multi-center Digitalis Investigation Group (DIG) trial dataset, undertaken to clarify digoxin’s effect on mortality and hospitalizations for worsening heart failure over a 37‑month follow-up period.

Methods

A survival analysis was conducted using the Digitalis Investigation Group (DIG) trial dataset. Patients with a left ventricular ejection fraction of 0.45 or less were randomly assigned to receive either digoxin (n=3,397) or placebo (n=3,403). Digoxin was administered at an average dose of 0.25 mg per day over a 37-month follow-up period. Kaplan-Meier survival curves and log-rank tests were used to evaluate time-to-event outcomes, and Cox proportional hazards regression was applied to derive risk ratios and 95% confidence intervals for mortality and hospitalization endpoints.

Results

Digoxin therapy demonstrated no statistically significant effect on overall mortality. Death occurred in 34.8% of the digoxin group (1,181 deaths) compared to 35.1% of the placebo group (1,194 deaths), and this difference is reported as a risk ratio (RR=0.99; 95% CI: 0.91 to 1.07; P=0.80) based on the observed event proportions in each arm. However, hospitalizations were reduced in the treatment group, with a significant reduction specifically in hospitalizations for worsening heart failure, presented as a risk ratio (RR=0.72; 95% CI: 0.66 to 0.79; P=0.001) derived from the cumulative proportions of patients hospitalized in the digoxin and placebo groups.

Conclusion

Digoxin did not improve overall survival but significantly reduced hospitalizations for worsening heart failure in patients with chronic heart failure and reduced ejection fraction. These findings reinforce digoxin’s role as an adjunct therapy focused on morbidity reduction and symptom stabilization, rather than as a primary mortality‑reducing agent, and provide additional context for its use alongside contemporary guideline‑directed heart failure treatments.

## Introduction

Digoxin is one of the most frequently recommended medications for the management of symptomatic heart failure [1]. Numerous short-term, randomized clinical trials have established that the withdrawal of digoxin precipitates a decline in patients' functional status, exercise capacity, and left ventricular ejection fraction. Despite these observed short-term symptomatic benefits, the comprehensive long-term impact of digoxin on overall mortality and specific hospital admissions remains poorly defined.

Heart failure remains a global public health crisis, affecting over 64 million people worldwide and serving as a leading cause of hospitalization and healthcare expenditure [2]. In the United States alone, the economic burden associated with heart failure is projected to rise significantly, driven largely by frequent readmissions [3]. In recent years, the clinical landscape has shifted toward novel neurohormonal therapies, such as angiotensin receptor-neprilysin inhibitors (ARNI) and SGLT2 inhibitors, which have proven highly effective [4]. Consequently, the utility of cardiac glycosides in contemporary clinical guidelines for treating patients with chronic heart failure is still widely debated [5]. Despite these advances, many patients remain symptomatic or are unable to tolerate newer agents, making it important to clarify whether digoxin continues to offer clinically meaningful reductions in worsening heart failure hospitalizations within the modern treatment landscape. To address this clinical knowledge gap, this study systematically re-examines the long-term effect of digoxin on mortality and on hospitalization for worsening heart failure (WHF) over a 37‑month period in a large randomized cohort, with a specific focus on clarifying digoxin’s morbidity‑reducing role within the context of contemporary heart failure therapies and guidelines.

## Materials and methods

Study design

This study is a retrospective secondary analysis utilizing the multi-center Digitalis Investigation Group (DIG) trial dataset [1]. The trial was a randomized, double-blind, placebo-controlled clinical study designed to evaluate the long-term effects of digoxin on mortality and morbidity. Given the large randomized trial sample size (N = 6,800) and the substantial number of deaths and hospitalizations observed over the 37‑month follow-up period, the analysis had adequate statistical power to detect clinically meaningful differences in mortality and worsening heart failure hospitalization between the digoxin and placebo arms. This secondary analysis uses a de‑identified, publicly available DIG trial dataset provided by the National Heart, Lung, and Blood Institute (NHLBI), and no direct interaction with participants or access to identifiable private information occurred.

Inclusion and exclusion criteria

The study included patients with a documented diagnosis of heart failure, characterized by a left ventricular ejection fraction (LVEF) of 0.45 or less. Participants were required to be in normal sinus rhythm. Patients were excluded if they had significant renal impairment or contraindications to digitalis therapy. The trial was conducted across clinical centers in the United States and Canada. These countries were selected a priori to provide a large, representative North American cohort treated within broadly comparable healthcare systems.

Data collection

The trial's planning and execution were overseen by a Steering Committee consisting of the National Heart, Lung, and Blood Institute (Bethesda, Maryland, USA), the Department of Veterans Affairs Cooperative Studies Program (Washington, D.C., USA), and regional cardiologists across the United States and Canada. Data points collected included baseline demographics, vital signs, and cause-specific hospitalization and mortality records evaluated over a mean follow-up period of 37 months.

Variables and covariates

The primary survival outcome of interest was defined as 'days till last follow-up or death,' which was treated as a continuous variable for survival time, and this was paired with the 'vital status of patient' (coded as 0=Alive, 1=Death) functioning as the censoring variable. Survival outcomes were predicted based on three primary predictor variables. The first was treatment assignment, recorded as 1 for digoxin treatment or 0 for placebo. The second was gender, which was evaluated as a potential confounding variable and recorded as 1 for male and 2 for female. Finally, morbidity, specifically 'hospitalization due to worsening heart failure' (WHF), was evaluated as a potential confounding variable and coded as 0 for no and 1 for yes. For the purposes of this analysis, hospitalization for worsening heart failure was modeled as a binary indicator of whether any such hospitalization occurred during the 37‑month follow‑up period, rather than as a time‑dependent covariate, to simplify interpretation of its association with overall survival.

Statistical analysis

All statistical and survival analyses were performed utilizing SAS software, version 9.4 (SAS Institute Inc., Cary, NC, USA). Survival curves were generated using the Kaplan-Meier estimator to visualize time-to-event data for days till last follow-up or death across treatment, gender, and hospitalization for worsening heart failure groups, in accordance with standard methods described by Kaplan and Meier [6]. The log-rank test was used to compare survival distributions among these stratified groups, and Cox proportional hazards regression models were applied to calculate risk ratios and 95% confidence intervals for mortality and hospitalization outcomes, following established survival analysis procedures outlined by Allison [7].

## Results

Baseline characteristics and descriptive statistics

The study population (N=6,800) was predominantly male (77.66%, n=5,281). At the end of the follow-up period, 4,425 patients (65.07%) were alive, while 2,375 patients (34.93%) had died. Stratification by treatment arm revealed no significant difference in vital status; the digoxin cohort experienced 1,181 deaths (representing 34.77% of the digoxin group) compared to 1,194 deaths (representing 35.09% of the placebo group) in the placebo arm (P=0.7815) (Table 1). Conversely, there was a highly significant difference in hospitalizations for WHF: 910 patients (26.79% of the digoxin group) in the treatment arm were hospitalized compared to 1,180 patients (34.68% of the placebo group) in the placebo arm (P<0.0001) (Table 1). The mean days until the last follow-up or death was similar between the placebo (1063.06 ± 459.92 days) and digoxin (1064.33 ± 450.60 days) groups (P=0.2324).

**Table 1:** Baseline characteristics and descriptive statistics Data are presented as number (%) unless otherwise indicated. SD, standard deviation; WHF, worsening heart failure.

Variable	Total (N = 6,800)	Placebo (n = 3,403)	Digoxin Treatment (n = 3,397)	P-value
Gender, n (%)				0.8235
Male	5,281 (77.66%)	2,639 (77.55%)	2,642 (77.77%)	
Female	1,519 (22.34%)	764 (22.45%)	755 (22.23%)	
Vital Status of Patient, n (%)				0.7815
Alive	4,425 (65.07%)	2,209 (64.91%)	2,216 (65.23%)	
Death	2,375 (34.93%)	1,194 (35.09%)	1,181 (34.77%)	
Hospitalization due to Worsening Heart Failure, n (%)				<0.0001
Yes	2,090 (30.74%)	1,180 (34.68%)	910 (26.79%)	
No	4,710 (69.26%)	2,223 (65.32%)	2,487 (73.21%)	
Days to last follow-up or death (days)				0.2324
Mean ± SD	1063.70 ± 455.25	1063.06 ± 459.92	1064.33 ± 450.60	

Kaplan-Meier survival estimates

**Figure 1:**
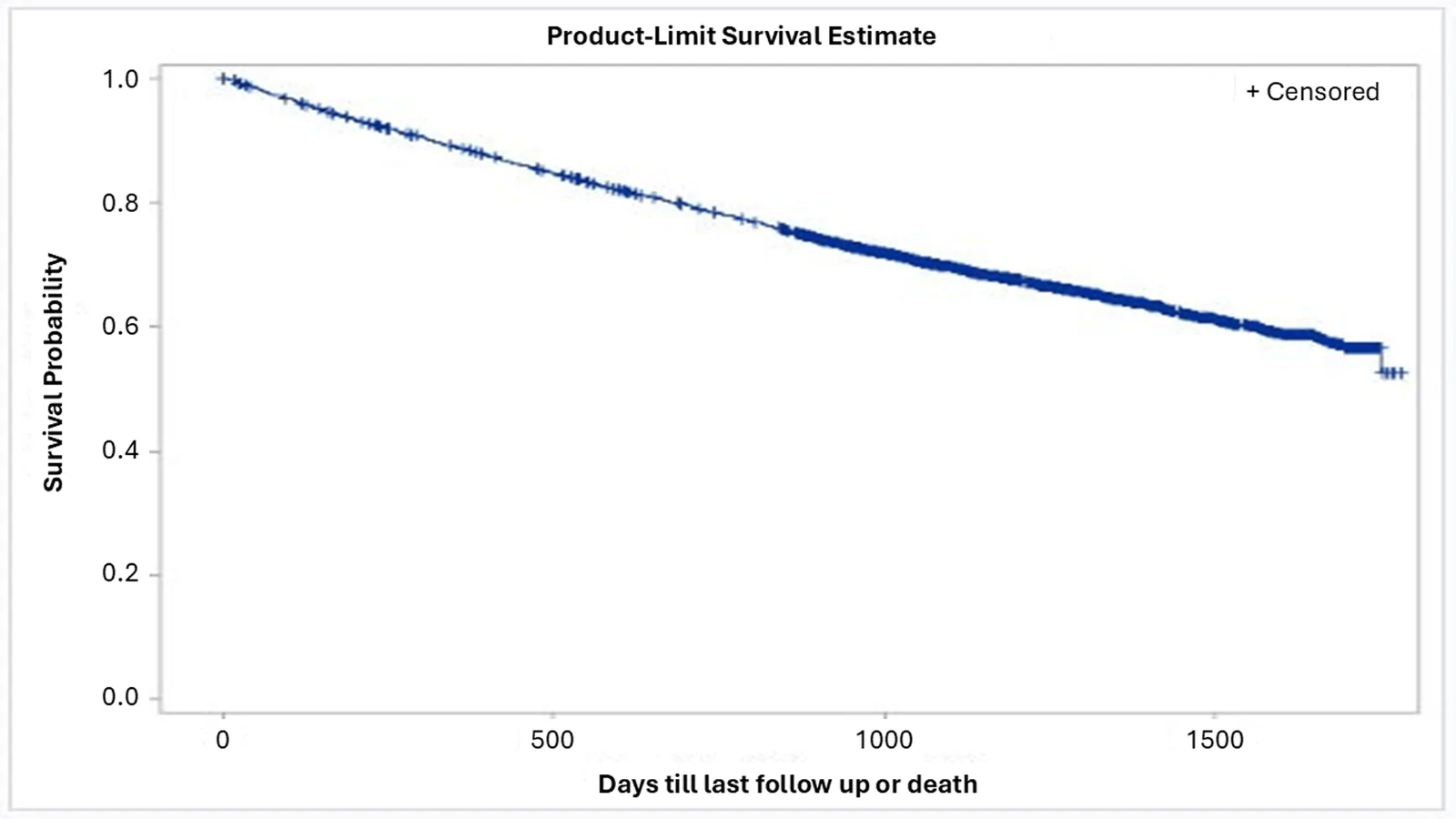
Overall Kaplan-Meier survival estimate for the entire study cohort The median survival time was not reached during the follow-up period, as overall survival remained above 50%.

**Figure 2:**
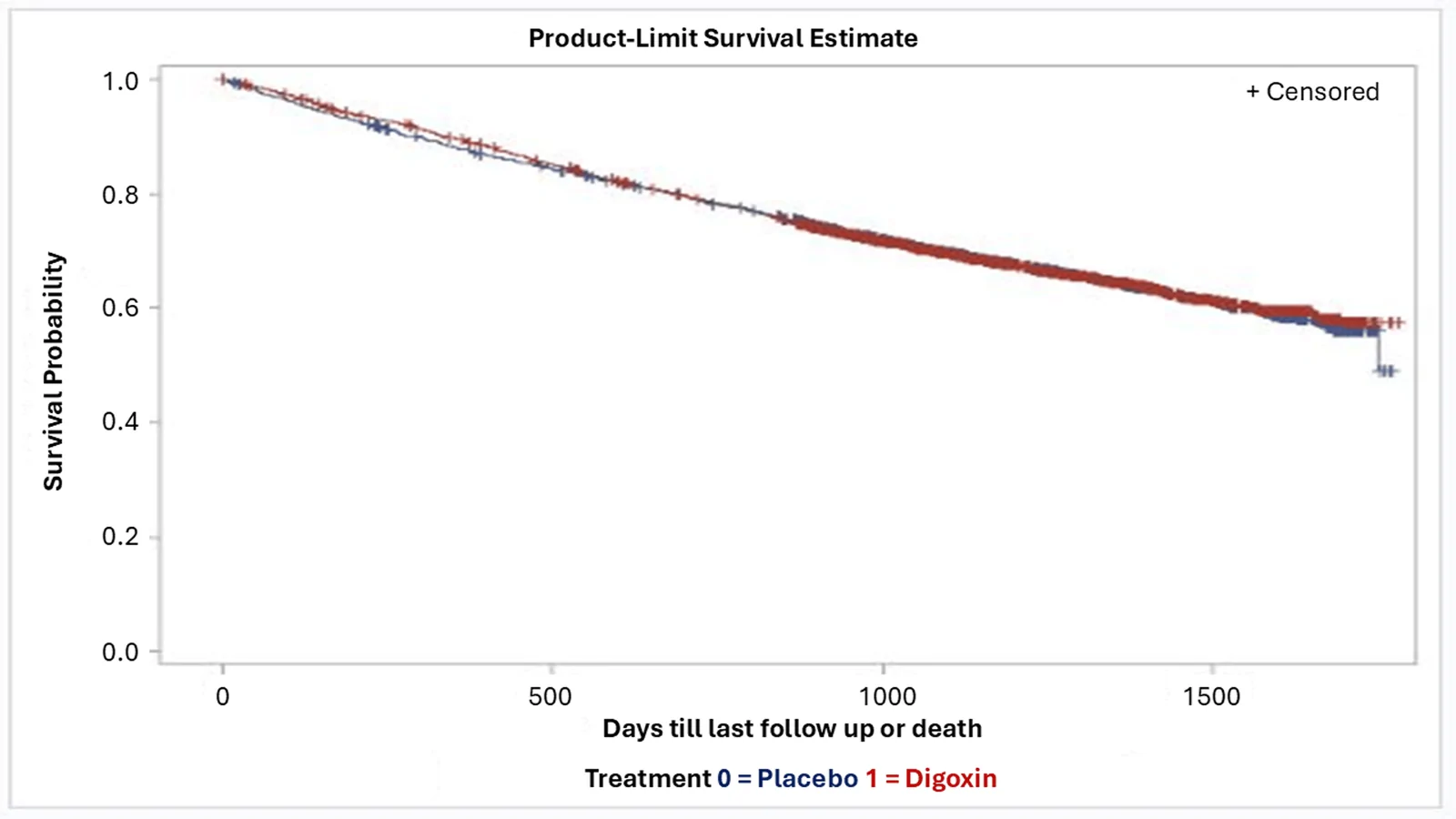
Kaplan-Meier survival estimates comparing digoxin treatment versus placebo The survival curves for digoxin and placebo were nearly identical, showing no significant difference in overall survival.

**Figure 3:**
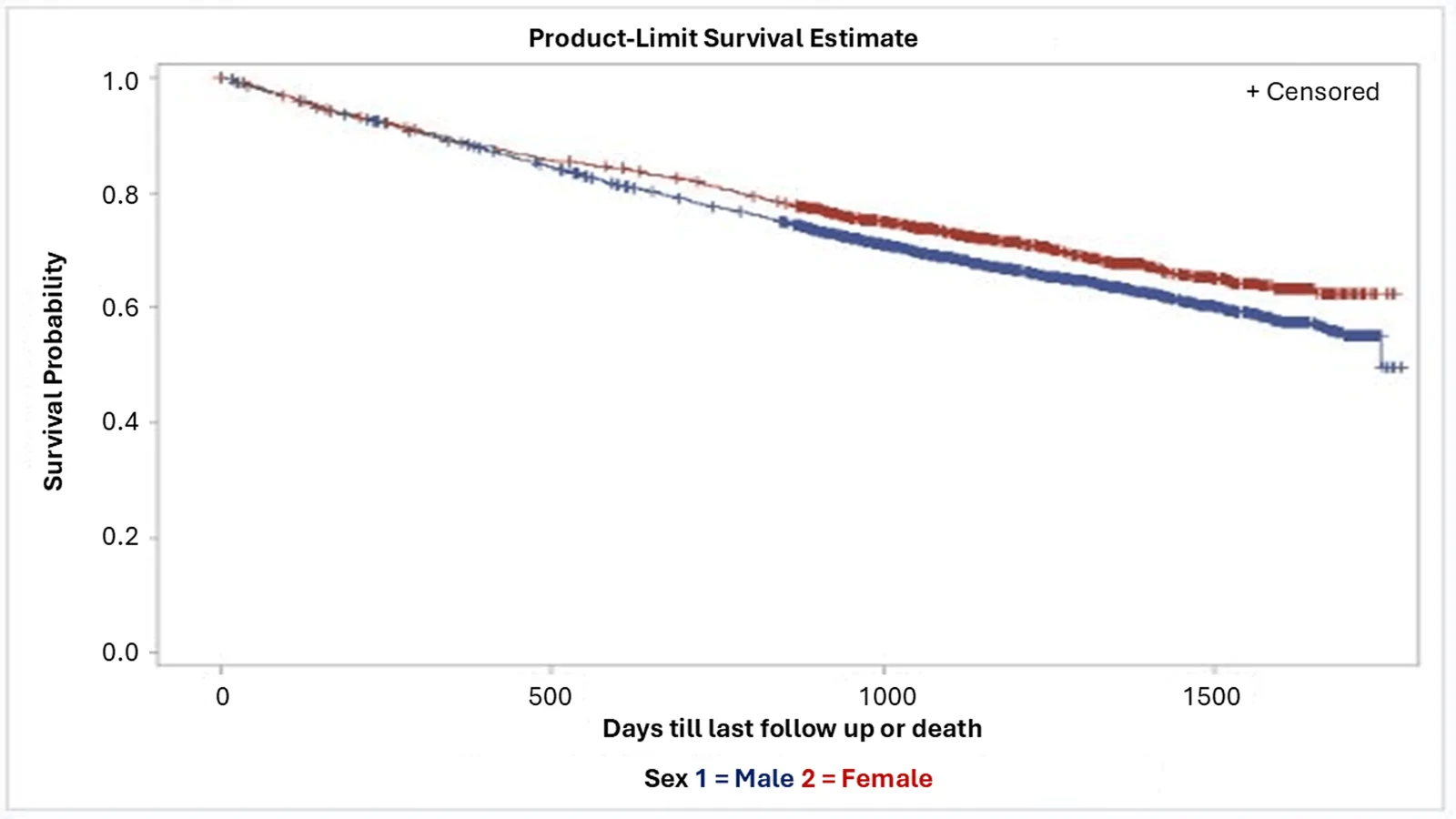
Kaplan-Meier survival estimates stratified by gender Probability was compared between male and female patients over the follow-up period.

**Figure 4:**
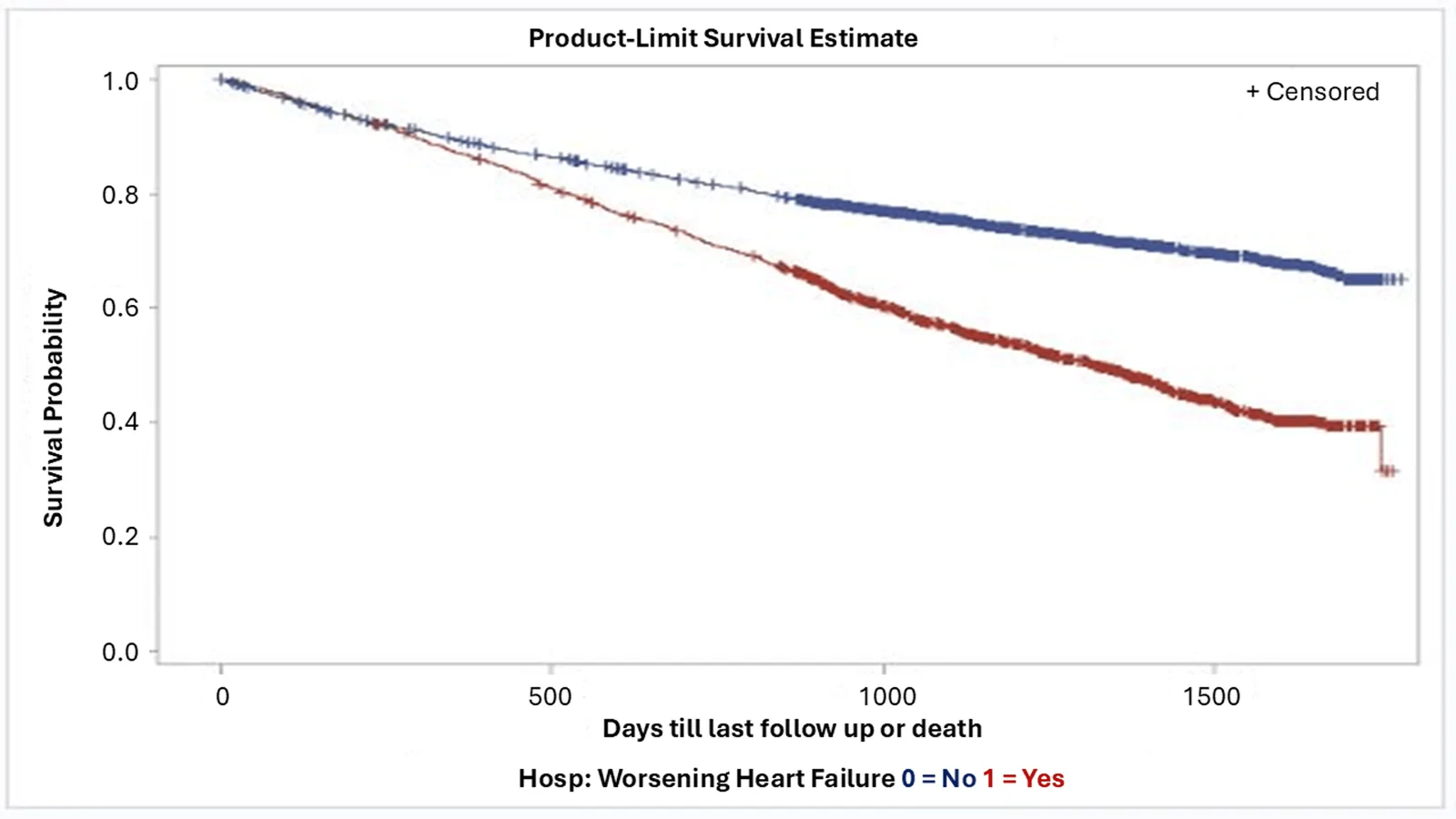
Kaplan-Meier survival estimates for patients with and without hospitalization for worsening heart failure (WHF) Patients hospitalized for worsening heart failure showed a markedly lower survival probability than those without WHF hospitalization.

The Kaplan-Meier survival curve for the entire sample did not reach a median survival time, as the overall survival probability never dropped below 50% (Figure 1). When stratified by treatment (Digoxin vs. Placebo), there was no median survival time reached for either group, and the survival curves were virtually identical (Figure 2). Similarly, when stratified by gender, neither the male nor female group reached a median survival time (Figure 3). However, when stratified by hospitalization for WHF, a stark contrast emerged (Figure 4). The median survival time for the hospitalization group (WHF=Yes) was approximately 1,320 days, whereas for the group without WHF hospitalization, no median length of stay was reached.

Univariate survival analysis

To statistically evaluate the visual findings from the Kaplan-Meier curves, log-rank and Wilcoxon tests were conducted. Regarding treatment, there was insufficient statistical evidence to reject the null hypothesis that the digoxin and placebo survival curves were the same (Log-rank P=0.8013; Wilcoxon P=0.8024). Conversely, regarding gender, there was statistically significant evidence to reject the null hypothesis that male and female survival curves were identical (Log-rank P=0.0009; Wilcoxon P=0.0026). Males experienced a slightly faster decline in survival probability over time compared to females. Finally, there was highly significant statistical evidence to reject the null hypothesis that the survival curves for those with and without WHF hospitalizations were the same (Log-rank P<0.0001; Wilcoxon P<0.0001). Survival probability dropped much more rapidly over time for those requiring hospitalization.

Multivariate survival analysis

A forward stepwise Wilcoxon test was utilized to evaluate the simultaneous covariate effects of treatment, gender, and WHF hospitalization on survival time. Hospitalization for WHF was added first and demonstrated a highly significant primary effect (Chi-square = 242.4; P<0.0001). Gender was added sequentially, yielding an incremental chi-square of 11.69 and an incremental P-value of 0.0006, providing sufficient evidence that gender independently impacts survival time. Treatment was added last, yielding an incremental chi-square of 0.95 and an incremental P-value of 0.3294. This value is not statistically significant (>0.05), confirming that digoxin treatment has no independent effect on overall survival time when accounting for WHF and gender. This forward stepwise Wilcoxon procedure served as the multivariate survival model complementing the Kaplan-Meier and Cox regression analyses described above.

## Discussion

Hospitalization for worsening heart failure was markedly reduced with digoxin treatment, and consequently, there were fewer deaths directly attributed to worsening heart failure in the digoxin cohort. These findings are directly consistent with the landmark Digitalis Investigation Group (DIG) trial, which established that digoxin significantly reduced the rate of hospitalizations for worsening heart failure without improving overall survival [1]. In addition, prior work has highlighted important sex-specific differences in heart failure outcomes, particularly between men and women [8]. A systematic review and meta-analysis by Vamos et al. (2015), encompassing 52 studies and over 621,000 patients, further confirmed this pattern: pooled risk ratios for death with digoxin were neutral in randomized controlled trials (RR = 0.99; 95% CI: 0.93-1.05), while digoxin was associated with a small but statistically significant reduction in all-cause hospital admissions across all study types (RR = 0.92; P < 0.001) [9]. These independent lines of evidence robustly support the present study's conclusion that digoxin's primary therapeutic value lies in morbidity reduction rather than mortality prevention.

The magnitude of hospitalization reduction observed in this analysis, a 26.79% worsening heart failure admission rate in the digoxin arm compared to 34.68% in the placebo arm, is consistent with findings from high-risk subgroup analyses of the DIG dataset. Ahmed et al. demonstrated that digoxin reduced the primary endpoint of all-cause and heart failure mortality or hospitalizations specifically among high-risk patients, including those with NYHA Class III-IV symptoms (HR = 0.65; P < 0.001) and LVEF < 25% (HR = 0.61; P < 0.001), an effect driven predominantly by the reduction in hospitalizations [10]. Similarly, a separate investigation of DIG trial data confirmed that digoxin's benefit in reducing heart failure hospitalizations is heterogeneous and most pronounced among patients at the highest predicted risk for heart failure-related admission [11]. These subgroup findings suggest that patient risk stratification may help identify those who stand to gain the most from adjunctive digoxin therapy.

Serum digoxin concentration (SDC) is an important modifier of clinical outcomes that warrants contextual discussion. Post-hoc analyses of the DIG trial have established that SDCs between 0.5 and 0.9 ng/ml were associated with lower mortality, all-cause hospitalization, and congestive heart failure-specific hospitalization, whereas SDCs exceeding 1.0 ng/ml were associated with increased mortality risk despite continued reductions in heart failure hospitalizations [10]. The DECISION (Digoxin Evaluation in Chronic Heart Failure: Investigational Study In Outpatients in the Netherlands) trial, which completed enrollment of 1,002 patients in 2023, is specifically designed to evaluate low-dose digoxin against a background of contemporary heart failure therapy, with cardiovascular mortality and total heart failure hospitalizations as the primary composite endpoint [12]. The results of this trial will be pivotal in clarifying whether digoxin's morbidity benefit is preserved at modern, lower doses.

These findings hold significant relevance in the context of evolving heart failure literature. Modern landmark therapies, such as angiotensin receptor-neprilysin inhibitors and sodium-glucose cotransporter-2 (SGLT2) inhibitors, have revolutionized care by successfully reducing both mortality and hospitalization [4]. A 2026 meta-analysis of 15 randomized controlled trials encompassing 28,484 participants demonstrated that SGLT2 inhibitors were associated with a 14% reduction in all-cause mortality (HR = 0.86; 95% CI: 0.79-0.92; P < 0.001) and a 26% reduction in heart failure hospitalization (HR = 0.74; 95% CI: 0.68-0.81; P < 0.001), with consistent benefits irrespective of ejection fraction or diabetes status [13]. Despite these advances, a significant proportion of patients remain symptomatic despite optimal guideline-directed medical therapy or cannot tolerate newer agents due to comorbidities such as renal impairment or hypotension. Our results reinforce the position of the 2022 AHA/ACC/HFSA guidelines, which maintain that digoxin remains a recommended, evidence-based adjunctive therapy for symptomatic patients specifically to reduce the risk of heart failure-related hospital admissions [5]. The utility of digoxin as a complement rather than competitor to contemporary neurohormonal agents represents a clinically pragmatic approach to the management of refractory heart failure symptoms.

Furthermore, our analysis revealed demographic disparities in heart failure prognoses, specifically finding that males are more susceptible to accelerated mortality over time than females [8]. This finding aligns with a propensity-matched analysis of DIG trial data by Ahmed et al., which demonstrated that while women with heart failure experienced reduced overall mortality compared to men, hospitalization rates were comparable between sexes [14]. Importantly, a seminal sex-based analysis of the DIG trial by Rathore et al. revealed a significant interaction between sex and digoxin therapy on mortality (P = 0.034): women randomized to digoxin had a higher rate of all-cause death than those receiving placebo (33.1% vs. 28.9%), whereas mortality rates were similar among men in both arms (35.2% vs. 36.9%) [15]. This aligns with broader cardiovascular literature indicating that the clinical profile and disease progression of heart failure can vary significantly between sexes, warranting highly personalized management strategies [8].

Ultimately, while digoxin does not extend total lifespan or lower total mortality, it successfully decreases the rate of hospitalization for worsening heart failure [1,9]. In the era of value-based healthcare, where reducing preventable readmissions is both a quality benchmark and a financial imperative, the sustained morbidity benefit of digoxin represents a tangible and clinically meaningful contribution. Future research, including the ongoing DECISION trial and the DIGIT-HF trial evaluating digoxin in advanced heart failure with reduced ejection fraction, will be essential in defining the precise role of cardiac glycosides within modern, individualized treatment paradigms [12].

## Conclusions

In conclusion, this study demonstrates that while digoxin does not improve overall survival in patients with chronic heart failure and reduced ejection fraction, it is highly effective at significantly reducing the rate of hospitalizations for worsening symptoms.

Our findings support previous evidence suggesting that digoxin’s principal clinical value lies in morbidity management and symptom stabilization, rather than as a primary mortality-reducing agent. In the modern healthcare landscape, where reducing frequent hospital readmissions and improving the day-to-day quality of life for patients are top clinical priorities, the role of digoxin in keeping patients out of the inpatient setting represents a critical and tangible benefit. By shifting the clinical evaluation from mere life extension to overall clinical stability, these findings provide a clearer, actionable description of digoxin’s ongoing function in the comprehensive management of chronic heart failure.

## References

[1] The Digitalis Investigation Group (1997). The effect of digoxin on mortality and morbidity in patients with heart failure. N Engl J Med.

[2] Ambrosy AP, Fonarow GC, Butler J (2014). The global health and economic burden of hospitalizations for heart failure: lessons learned from hospitalized heart failure registries. J Am Coll Cardiol.

[3] Virani SS, Alonso A, Aparicio HJ (2021). Heart Disease and Stroke Statistics-2021 Update: a report from the American Heart Association. Circulation.

[4] McMurray JJ, Packer M, Desai AS (2014). Angiotensin-neprilysin inhibition versus enalapril in heart failure. N Engl J Med.

[5] Heidenreich PA, Bozkurt B, Aguilar D (2022). 2022 AHA/ACC/HFSA Guideline for the Management of Heart Failure: a report of the American College of Cardiology/American Heart Association Joint Committee on Clinical Practice Guidelines. Circulation.

[6] Kaplan EL, Meier P (1958). Nonparametric estimation from incomplete observations. J Am Stat Assoc.

[7] Allison PD (2010). Survival Analysis Using SAS: A Practical Guide. SAS Institute Inc, Cary, NC.

[8] Hsich EM, Piña IL (2009). Heart failure in women: a need for prospective data. J Am Coll Cardiol.

[9] Vamos M, Erath JW, Hohnloser SH (2015). Digoxin-associated mortality: a systematic review and meta-analysis of the literature. Eur Heart J.

[10] Ahmed A, Rich MW, Love TE (2006). Digoxin and reduction in mortality and hospitalization in heart failure: a comprehensive post hoc analysis of the DIG trial. Eur Heart J.

[11] Upshaw JN, van Klaveren D, Konstam MA, Kent DM (2018). Digoxin benefit varies by risk of heart failure hospitalization: applying the Tufts MC HF risk model. Am J Med.

[12] van Veldhuisen DJ, Rienstra M, Mosterd A (2024). Efficacy and safety of low-dose digoxin in patients with heart failure. Rationale and design of the DECISION trial. Eur J Heart Fail.

[13] Mao X, Liu W, Hu B, Xu E (2026). Glucose cotransporter-2 inhibitors on mortality and hospitalization in heart failure patients: a comprehensive meta-analysis. Front Endocrinol (Lausanne).

[14] Ahmed MI, Lainscak M, Mujib M (2011). Gender-related dissociation in outcomes in chronic heart failure: reduced mortality but similar hospitalization in women. Int J Cardiol.

[15] Rathore SS, Wang Y, Krumholz HM (2002). Sex-based differences in the effect of digoxin for the treatment of heart failure. N Engl J Med.

